# RETRACTION: Ivermectin Synergizes Sorafenib in Hepatocellular Carcinoma via Targeting Multiple Oncogenic Pathways

**DOI:** 10.1002/prp2.70222

**Published:** 2026-02-13

Lu H. Zhou L. Zuo H. Le W. Hu J. Zhang T. Li M. Yuan Y. “Ivermectin Synergizes Sorafenib in Hepatocellular Carcinoma via Targeting Multiple Oncogenic Pathways.” *Pharmacology Research & Perspectives* 10 no. 3 2022: e00954. https://doi.org/10.1002/prp2.954.

The above article, published online 14 May 2022 on Wiley Online Library (wileyonlinelibrary.com), has been retracted by agreement between the authors, the Editor‐in‐Chief Professor Jennifer Martin, the British Pharmacological Society and John Wiley and Sons Ltd. The retraction has been agreed after concerns were raised that the Corresponding Author was not personally involved in the submission process, did not sign the Open Access Agreement, and did not review or approve the final version of the manuscript prior to submission. Additionally, the investigation uncovered evidence of duplication of several images and figures from previously published articles. The authors state that the conclusions of the article are otherwise unaffected.

